# Plastic Type and Condition Have Minimal Impact on Associated Marine Biofilm Communities

**DOI:** 10.1111/1462-2920.70214

**Published:** 2025-12-12

**Authors:** J. A. Wallbank, J. M. Kingsbury, O. Pantos, L. Weaver, D. A. Smith, M. Barbier, B. Theobald, V. Gambarini, G. Lear

**Affiliations:** ^1^ School of Biological Sciences University of Auckland Auckland New Zealand; ^2^ Institute of Environmental Science and Research Christchurch New Zealand; ^3^ Scion, Te Papa Tipu Innovation Park Rotorua New Zealand

**Keywords:** biodegradation, heterochain polymer, homochain polymer, plastisphere, polyamide

## Abstract

The ecological impacts of plastics and their additives on marine microbiota remain unclear. We applied prokaryotic 16S rRNA gene and fungal ITS2 region amplicon sequencing, alongside shotgun metagenomic sequencing, to identify compositional and functional changes in microbial communities on marine plastic. Five common plastics, both non‐aged and artificially aged, were submerged in Auckland Harbour, Aotearoa‐New Zealand. Biofilms on linear low‐density polyethylene (LLDPE), nylon‐6 (PA), polyethylene terephthalate (PET), polylactic acid (PLA), oxo‐biodegradable LLDPE (OXO) and glass were sampled over 12 months. The taxonomy and functional potential of biofilm communities differed from surrounding seawater communities and varied with biofilm age. Younger biofilms were more diverse, with Proteobacteria, unknown fungi and unclassified Metazoa dominating prokaryotic, fungal and eukaryotic communities, respectively. Taxa related to previously reported plastic‐degraders were found in very low abundance across all substrates. Plastic type and UV‐ageing did not significantly shape biofilm communities over a year. Although some genes differed in relative abundance due to UV‐ageing, overall functional profiles remained consistent across plastics. Genes conferring reported plastic‐degrading traits were present regardless of plastic type, UV‐ageing and biofilm age. Nevertheless, nylon hydrolases were notably associated with PA, suggesting marine plastic impacts may be restricted to taxa or functions involved in its degradation.

## Graphical abstract

Plastics serve as a novel surface for microbial colonisation, yet their ecological functioning remains poorly understood. Here, we characterise microbial communities colonising plastic in the marine environment over time using a multi‐omics approach, highlighting previously reported plastic‐degrading taxa and the degradation potential within these communities.

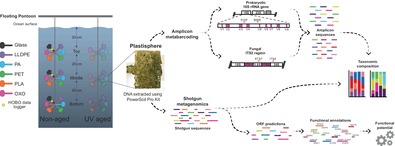

## 1 Introduction

Worldwide, over 400 million tons of recalcitrant plastic material is produced annually, with approximately 50% being single‐use (Geyer et al. 2017; Schnurr et al. 2018). Improper disposal has led to 1.1 to 4.9 million tonnes of plastic debris in our oceans (Eriksen et al. 2023). Microbial communities rapidly colonise these plastics, forming a ‘plastisphere’ (Zettler et al. 2013). Investigations have focused on identifying bacteria that colonise different marine plastics, their potential to degrade them, and observing differences among bacterial communities based on their location and age (Dussud et al. 2018; Kirstein et al. 2019; Oberbeckmann et al. 2016; Wallbank et al. 2022). However, Archaea, eukaryotes and fungi (Kettner et al. 2017; Kirstein et al. 2018; Lacerda et al. 2022) within the plastisphere remain understudied. Further exploration of these taxa could provide a broader understanding of the ecological impacts of marine plastic debris.

Bryant et al. (2016) found distinct taxa associating with plastics, and through the study of metagenomics data, confirmed genes associated with cell‐to‐cell interactions, chemotaxis and nitrogen fixation were enriched in plastisphere communities, as compared to those in ambient marine water. Later studies have shown the plastisphere to act as a reservoir for genes conferring polymer biodegrading potential (Jahanshahi et al. 2023) and to be an important substrate for genetic material exchange (Arias‐Andres et al. 2018; Wu et al. 2022). These findings highlight the potential of bioprospecting plastisphere‐associated communities for novel polymer‐degrading genes.

To address the limitation of prior studies on plastisphere microbiomes, we combined metabarcoding and shotgun metagenomic methods to determine the taxonomic and functional diversity of microbial communities on five plastic polymers (compared to an inert glass control, and to the communities present within the surrounding water) in the marine environment of a commercial port, over 52 weeks. We focused on common marine plastics, including linear low‐density polyethylene (LLDPE), commonly used for disposable bags, liners and wraps, nylon‐6 (PA), which is widely used for plastic ropes and fishing nets, and polyethylene terephthalate (PET), the most common plastic for making drink bottles. Polylactic acid (PLA) and oxo‐biodegradable linear low‐density polyethylene (OXO) were also included as degradable polymers. These five plastics encompass diverse molecular structures, with LLDPE and OXO having homoatomic carbon–carbon backbones. In contrast, PA, PET, and PLA contain noncarbon heteroatoms such as nitrogen (N) or oxygen (O) in the polymer backbone, which may promote their degradation (Lear et al. 2022).

We hypothesised that each plastic polymer would host specific microbial communities due to the differing chemical compositions of the polymer backbones (*polymer H*_1_). We also predicted that younger biofilms would be more diverse and distinct to each plastic type due to the closer average proximity of microbes to the plastics' surface (*temporal H*_1_). To replicate environmental UV weathering, we included two conditions for each plastic polymer: virgin polymer and artificially UV‐aged plastic (herein referred to as ‘non‐aged’ and ‘aged’). We hypothesised that aged plastics would host more diverse and plastic‐specific microbial communities and functions due to their increased surface roughness and the presumed greater accessibility of short carbon chains resulting from their prior UV photodegradation (*ageing H*_1_).

## 2 Materials and Methods

### 2.1 Sampling Site and Collection

**FIGURE 1:**
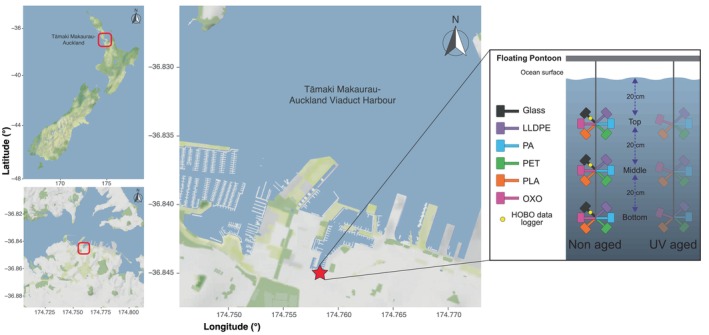
Geographical location and schematic of in situ plastic and glass deployment on a stainless steel structure in the Lighter Basin Marina, Tāmaki Makaurau‐Auckland Viaduct Harbour, Aotearoa‐New Zealand. The five plastic types in the water column, including aged and non‐aged plastics, were affixed to separate poles along with glass control samples. Samples and data loggers were placed at depths of 20, 40 or 60 cm from the water's surface. Geographic maps were constructed with the ‘ggmap’ package (version 4.0.0; Kahle and Wickham 2013) in R (version 4.2.1; R Core Team 2023).

Our deployment structure was situated on a floating pontoon in the Lighter Basin Marina, Tāmaki Makaurau‐Auckland Viaduct Harbour, Aotearoa‐New Zealand (A‐NZ) (36°50′42.5″S; 174°45′30.0″ E; Figure 1). Deployment occurred on 16 March 2020 (austral autumn), with sampling at 13, 26, 39 and 52 weeks after deployment to test our temporal *H*_*1*_, hereafter referred to as months 3, 6, 9 and 12.

To test the polymer *H*_1_, as in Laroche et al. (2023), substrate types used in the study were glass, LLDPE, OXO, PA, PET and PLA (Table S1) moulded into rectangular paddles (75 mm by 50 mm by 3 mm) with an integrated arm. To test our ageing *H*_1_, artificially aged plastics were used alongside non‐aged plastic paddles. The natural weathering process usually experienced by plastics in the ocean was mimicked using a modified ASTM D4329‐13/ASTM G154 test (ASTM International 2023) to apply repeated cycles of 8 h of ultraviolet light (preferred irradiance at 340 nm was 0.89 W/m^2^) at 50°C, with a final exposure to condensation for 4 h (to mimic dew) at 50°C. While this method may not precisely mimic the variable conditions to which marine plastics are usually exposed, it is the international standard practice for fluorescent ultraviolet (UV) lamp exposure of plastics. Paddles were moved across the device weekly and flipped after 400 h to ensure even exposure, remaining within the device for a further 400 h. Plastic paddles and glass slides were distributed and oriented horizontally in the marine water as previously described (Wallbank et al. 2022), with non‐aged and aged plastics on separate stainless‐steel poles. Poles were constantly submerged, with one of each substrate spaced 20, 40 and 60 cm from the water's surface, hereby referred to as top, middle and bottom paddles. At 15‐min intervals throughout the experiment, data loggers (HOBO Pendant MX2202; Onset Computer Corp, Bourne, MA, USA) attached to the structure at each depth recorded light intensity and temperature within the water column.

As per Wallbank et al. (2025), triplicate plastic paddles were collected for each substrate type (one per depth) alongside triplicate glass slides. Glass slides are commonly adopted as control substrates in studies of microbial plastic degradation since microorganisms are unable to use glass as a carbon or energy source. Therefore, including glass control substrates helps ascertain if microbial community attributes observed on the plastic substrates are due to plastic degradation or toxicity, compared to more generalist features associated with surface‐attached marine communities. At each sampling time, three field sampling controls were collected by opening empty sample bags on site. In parallel, three 2 L seawater samples were collected at each sampling time from the exact location and approximate depth of each deployment structure to determine ambient seawater microbial community compositions. All samples were placed on ice for transportation back to the laboratory for processing (i.e., 3 depths [top, middle bottom] x 5 plastic substrates [LLDPE, OXO, PA, PET and PLA] × 2 conditions [non‐aged, aged] + 3 seawater samples +3 glass biofilm samples +3 ‘field blank’ samples = a total of 39 samples per sampling date). Two plastic paddles were lost from the structure between 9 and 12 months. Since there were four sampling dates (i.e., at 3, 6, 9 and 12 months), 154 samples were collected in total.

### 2.2 Sample Processing

**FIGURE 2:**
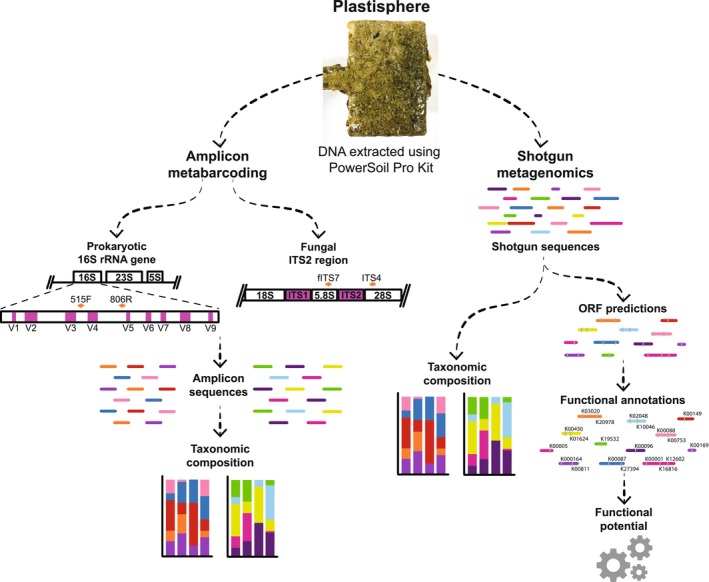
Amplicon metabarcoding and shotgun metagenomic sequencing approaches used in this study. Conserved and hypervariable regions of the 16S rRNA gene and the ITS region are shown by white and purple boxes, respectively. The orange arrows represent the approximate positions of the primers used.

As previously described, biomass was extracted from the plastic paddles and glass slides within 2 h of collection (Laroche et al. 2023; Wallbank et al. 2022); seawater was filtered to collect microbial biomass onto 0.2 μm filter membranes. DNA was manually extracted from up to 250 mg of biomass from each pelleted biofilm and seawater filter sample using DNeasy PowerSoil Pro kits (Qiagen, Hilden, Germany), as described in Wallbank et al. (2022). Microbial DNA was extracted from a total of 154 samples as previously outlined (i.e., 130 biofilm samples, 12 seawater samples and 12 field blank samples), along with a mock community (ZymoBIOMICS Microbial Community Standard) to identify any bias in our DNA extraction method. While the extraction of DNA using methods such as DNeasy kits is acknowledged to underestimate the abundances of taxa with stronger cell walls [e.g., Gram‐positive bacteria (Iturbe‐Espinoza et al. 2021) and fungal cell walls strengthened with substances including melanin and chitin (Sikarware and Tenguria 2025)], we chose to use this approach based on the best‐practice recommendations of Lear et al. (2018). DNA was then stored at −20°C until required for metabarcoding or shotgun sequencing (Figure 2).

### 2.3 PCR And DNA Amplicon Sequencing

As described in the Supporting Information: Methods, PCRs were performed to amplify the prokaryotic 16S rRNA gene fragments and fungal ITS2 regions of samples using universal primers (prokaryotic 515F and 806R; fungal fITS7 and ITS4; Table S2) containing Illumina adapter sequences (Kozich et al. 2013). Mock microbial standards (ZymoBIOMICS Microbial Community and DNA standards) and negative controls with no DNA added were included in every reaction run. Samples were sent to the Auckland Genomics Facility (The University of Auckland, New Zealand) for library preparation and sequencing on an Illumina MiSeq instrument using 2‐by‐300‐bp V3 chemistry.

### 2.4 Shotgun Sequencing

DNA extracts from three, nine and 12‐month samples were selected for shotgun metagenomic sequencing to explore the functional potential of the plastisphere microbial communities over time. A total of 106 samples (i.e., 117 samples—9 ‘field blank’ samples—2 missing samples = 106 samples) were sent to the Otago Genomics Facility (The University of Otago, A‐NZ) on ice to prepare Thruplex DNA libraries using NextSeq 2000 P3‐300 reagent kits (Illumina Inc., CA, USA). DNA sequencing was conducted on an Illumina NextSeq 2000 instrument using 2‐by‐150‐bp paired‐end chemistry.

### 2.5 Accession Numbers

Raw amplicon sequence reads and unassembled metagenomic sequence data are available from the NCBI Sequence Read Archive (SRA) under BioProject: PRJNA1163745 (Reviewer link—https://dataview.ncbi.nlm.nih.gov/object/PRJNA1163745?reviewer=79qb7789ic6tslg4e1tjqmn5iv).

### 2.6 Processing of DNA Sequence Data

DNA sequence reads were processed as described in the Supporting Information: Methods. For analysis of DNA amplicon data, this means that reads were processed using the DADA2 pipeline to identify amplicon sequence variants (ASVs); taxonomy was assigned using the prokaryotic 16S rRNA gene SILVA reference database (version 138.1; McLaren and Callahan 2021) and the UNITE ITS reference database to identify fungal‐derived DNA sequences (general FASTA release version 9.0 (18.07.2023); Abarenkov et al. 2023). ASVs were filtered to remove reads assigned as mitochondria or chloroplasts and retain only those designated as Archaea, Bacteria or Fungi. Taxonomic classifications of unassembled reads were obtained against the SILVA database using Metaxa2 (version 2.2.3; Bengtsson‐Palme et al. 2015).

### 2.7 Functional Assignment of Metagenomic Sequence Data

The ‘fq2fa’ function of IDBA‐UD (version 1.1.3; Peng et al. 2012) was used to merge forward and reverse‐filtered unassembled metagenomic reads into one file per sample. Open reading frames (ORFs) of unassembled reads were predicted using FragGeneScan (version 1.31; Rho et al. 2010). Analyses using Prodigal (version 2.6.3; Hyatt et al. 2010) yielded highly similar results. The sequence aligner DIAMOND (version 2.1.6; Buchfink et al. 2015) was used to annotate predicted genes against reference databases. To investigate and predict patterns regarding the functional potential of the microbial communities, any ORFs with functional content were determined against a database containing non‐redundant proteins from NCBI (accessed 03.11.2023; Benson et al. 2018) and SEED databases (Overbeek et al. 2014) via MEGAN6 (version 6.25.10; Huson et al. 2016). Biological pathways were determined using the KEGG database (version 07.04.2024; Kanehisa and Goto 2000), retaining only reads with at least 50% amino acid sequence identity, 70% query coverage, an e‐value of less than 1e^−5^, and a minimum read count of one. Predicted amino acid sequences were also aligned to enzymes with presumed plastic degradation potential within a curated database that contains genes putatively conferring plastic degradation (plasticDB.org; Gambarini et al. 2022), and only reads with 70% amino acid sequence identity, 70% query coverage and an e‐value of less than 1e^−5^ were retained for downstream analysis.

### 2.8 Preparation of Sequence Data

All statistical and quantitative analyses and data visualisation were performed using R (version 4.2.1; R Core Team 2023). Detailed statistical and quantitative analyses are provided in the Supporting Information: Methods and have been previously described by Wallbank et al. (2025).

## 3 Results

Light intensity differed significantly among sampling times (ANOVA; *p* = 0.009), ranging from a daily average illuminance of 1177 to 12,316 lux (Table S3). Illuminance among depths was significantly different when accounting for variation at each sampling date (standard linear mixed modelling (LMM), *p* = 0.013). Confidence intervals confirmed significantly greater illuminance of the top paddles, whereas illuminance was not significantly different when comparing the middle and bottom sampling layers. Average water temperature differed significantly with time (ANOVA; *p* < 0.001), being highest, on average, in March (22.3°C) and lowest in June (14.4°C; Table S3). No significant difference was observed among sampling depths (ANOVA; *p* = 0.998; LMM *p* = 0.219).

### 3.1 Sequencing Outputs

Quality filtering removed negative control data, leaving 26,747 and 9676 ASVs for prokaryotic and fungal analysis, respectively; two samples for fungal analysis were removed due to having no reads after filtering. Shotgun metagenomic data yielded an average of *c*. 9 million reads per sample after filtering read quality. Of the metagenomic reads, a total of 52,875,194 reads were classified into 764 SEED functions, while 310,510,353 reads were classified into 19,856 KEGG Orthology groups and 38,091 reads were assigned to 42 previously reported plastic‐degrading microbes. Results from the analysis of mock community DNA were largely as expected and are provided in the Supporting Information (Figure S1). No fungi within the mock community DNA standards were identified as *Saccharomyces*; only *Cryptococcus* was identified, as previously reported by Wallbank et al. (2025). Following DADA2 processing, no reads were retained for fungal mock community extractions, indicating a potential extraction and primer bias against fungal taxa that are hard to lyse, with a potential mismatch against Ascomycota and Basidiomycota (Ihrmark et al. 2012; Taylor et al. 2016).

### 3.2 Diversity and Composition of Prokaryotic and Fungal Communities Across Sample Depths, Ages, Substrate Type and Condition

Overall, the ASV richness and evenness of marine plastisphere communities were mainly influenced by biofilm age, with depth and substrate type having less impact (Figure S2 and Table S4). All three alpha diversity indices were significantly higher in the relative diversity, evenness and richness of prokaryotic taxa in the youngest samples (3 months) than for samples collected at all other times (6, 9 and 12 months). Chao1 estimates were highest at 3 months (634.08) compared to the later months (280.68–376.44), with a similar trend observed for Shannon estimates (3 months = 5.76 vs. 6–12 months = 4.97–5.29), and InverseSimpson (3 months = 242.17 vs. 6–12 months = 74.88–164.22). No overall significant difference in community composition or relative diversity was observed due to plastic condition (i.e., exposure to UV light). However, a significant interaction was found between sampling age and condition on the relative diversity of the prokaryotic communities (Chao1 and Inverse Simpson indices; Table S4). At 3 months, non‐aged and aged plastics had significantly richer communities than non‐plastic substrates (pairwise Wilcoxon rank sum; Chao1 *p* < 0.001, all other pairwise comparisons *p* > 0.05). Although prokaryotic communities on non‐aged and aged plastics at 9 months were not significantly different (pairwise Wilcoxon rank sum; InvSimpson *p* = 1.00), they were significantly less diverse than at all other times (pairwise Wilcoxon rank sum; InvSimpson *p* < 0.044).

**FIGURE 3:**
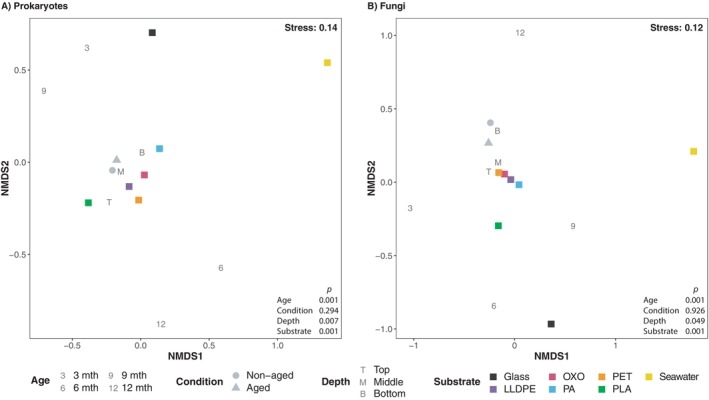
Non‐metric multidimensional scaling (NMDS) plots derived from Bray–Curtis dissimilarity matrices of ASV data. ASV data were obtained from microbial communities in seawater and biofilms on glass, LLDPE, OXO, PA, PET and PLA deployed in the marine environment for up to 12 months. Each point on plots (A) and (B) represents the average data per variable (i.e., ‘T' represents an average of data from all biofilm samples collected at the shallowest depth).

All subsequent analyses used CSS‐transformed amplicon data to correct for differences in sampling depth while retaining the maximum amount of data. Multidimensional analyses of biofilm microbial community data showed separate groupings compared to seawater communities (Figure 3), as reflected by pairwise PERMANOVA of the data (prokaryotes and fungi, seawater vs. plastic substrates, both *p* = 0.001; Figure S3A,B). After removing seawater and glass community data, substrate type and biofilm age were significantly related to changes among plastisphere microbial communities (prokaryotes and fungi; PERMANOVA; *p* = 0.001, pairwise PERMANOVA *p* = 0.001 for all comparisons; Figures 3 and S3A–D). However, we found no significant pairwise differences in bacterial or fungal community composition among the plastic polymers (Figure S3A,B). Together, these findings lead us to reject polymer *H*_*1*._ Betadisper analyses suggested that variations in data dispersion among all substrate types (i.e., including glass and seawater) may result in significant PERMANOVA results (Figure S3E,F). Larger mean Bray‐Curtis distances among biofilm data (prokaryotes—between 0.923 (OXO) and 0.940 (PA); fungi—between 0.854 (PA) and 0.888 (LLDPE)) indicated greater dispersion in comparison to the seawater data (0.787 and 0.813 for prokaryotes and fungi, respectively). Biofilm age was significantly correlated to betadisper values (betadisper; biofilm age *p* = 0.001; Figure S3E,F); thus, related PERMANOVA results may be due to the increased dispersion of data at six and 12 months. Therefore, while we predicted that younger biofilms would be more distinct to each plastic type, we witnessed no significant reduction in data dispersion as samples aged (i.e., community data from samples associated with different plastics did not converge over time, which would indicate the formation of more generalist communities over time); this leads us to reject ‘temporal *H*_*1*_’.

The artificial weathering of plastic substrates did not appear to cause compositional differences among microbial plastisphere communities (Figure 3), as supported by pairwise PERMANOVA (Figure S3A; prokaryotes *p* ≥ 0.057, Figure S3B; fungi *p* ≥ 0.240). Furthermore, the condition of plastics did not significantly impact the relative taxonomic diversity or richness of their associated communities (Table S4), causing us to reject our ageing *H*_1_. Sample depth was significant for biofilm prokaryotic and fungal community compositions (PERMANOVA; prokaryotes *p* = 0.007 and fungi *p* = 0.049; Figure 3). Prokaryotic and fungal communities on the top paddles differed significantly from those on the bottom, yet no significant variation in data dispersion between different biofilm depths was observed (Figure S3C,D).

The 16S rRNA gene and ITS2 region data from shotgun sequencing statistically supported the findings generated by DNA amplicon sequence analysis (Figure S4). Moderate to weak positive significant correlations were observed between the Bray–Curtis dissimilarity matrices from prokaryotic and fungal communities comparing amplicon and metagenomic data (Mantel test using Spearman's rank correlation coefficient; prokaryotes *r* = 0.273, *p* = 0.001; fungi *r* = 0.052, *p* = 0.041).

### 3.3 Taxonomies of Plastisphere‐Associated Microorganisms

Metagenomic taxonomic data were investigated to determine an average proportional distribution of each biological domain (Bacteria, Archaea, and Eukarya) within our samples. Taxonomically assigned short subunit (SSU) and long subunit (LSU) rRNA gene fragments within metagenomic reads were mainly comprised of Eukaryotes (78.36%), with Bacteria and Archaea comprising the remaining 21.60% and 0.04%, respectively (Figure S5A).

**FIGURE 4:**
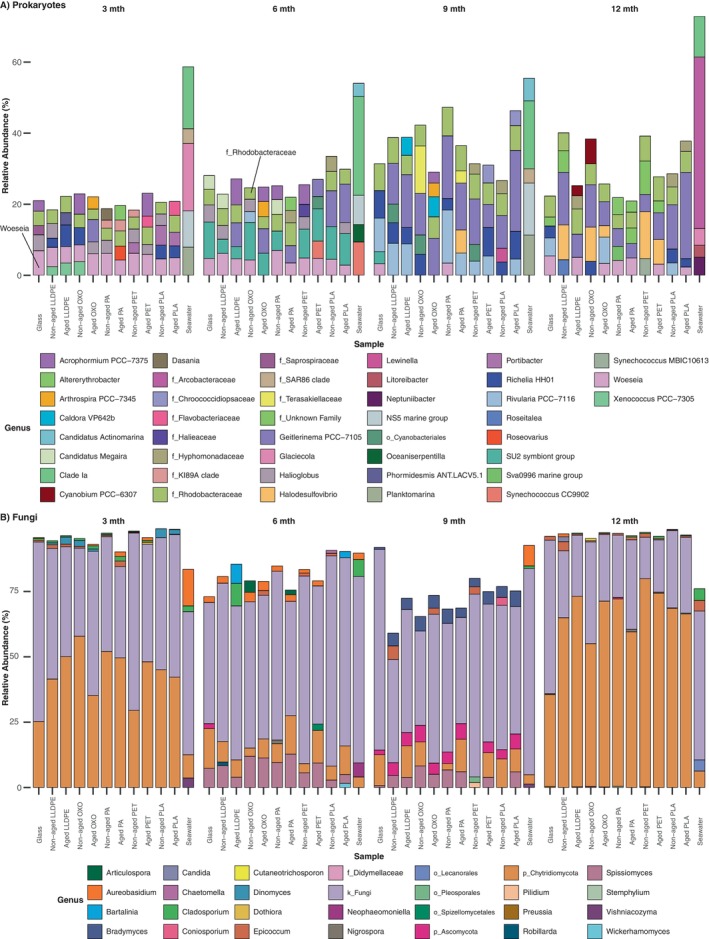
Relative abundance (%) of the five most dominant (A) prokaryotic and (B) fungal genera present in microbial communities on glass, plastics and in seawater, collected at three‐month intervals over 1 year. Averages were calculated separately across sampling depths for each substrate. A unique colour represents each genus. Taxa unassigned at the genus level were assigned their last known taxonomic rank, denoted by ‘_’ (i.e., k_fungi represents kingdom_fungi).

In further support of polymer *H*_*1*_, we found no evidence for an effect of plastic polymer type on the taxonomic composition of their associated microbial communities, assigned using unassembled metagenomic reads (Figure S4). Regardless of the sequencing approach, Proteobacteria dominated prokaryotic communities associated with plastic substrates, with Cyanobacteria increasing in relative abundance in biofilm communities up to 9 months, with a lower abundance present at 12 months (biofilm age ANOVA *p* < 0.001; Tukey HSD *p* < 0.002; Figures S5B and S6A). ASVs associated with the family Rhodobacteraceae and genus *Woeseia* were among the five most abundant prokaryotic genera per sample within biofilm communities on most plastics and glass (Figure 4A). Archaeal phyla were detected in very low abundance within our amplicon and metagenomic samples (Figures S5A,C and S6A). Of the metagenomic archaeal reads, Thaumarchaeota was the main phylum dominating the archaeal plastisphere communities at 3 months, with Euryarchaeota found across most biofilm communities and Crenarchaeota present in OXO, aged PET and all PLA biofilms, with a lower abundance on non‐aged PA and within seawater samples at this time (Figure S5C). The fungal communities are predominantly comprised of unknown fungi (k_fungi) and taxa assigned to the phylum Chytridiomycota, regardless of substrate type, plastic condition or sampling time (Figures 4B and S6B). *Aureobasidium*, *Cladosporium* and *Epicoccum* sp. were abundant on plastic and glass at every biofilm age. While certain genera were more abundant on specific plastics, their abundance was still low compared to the unknown fungal genera. Of the eukaryotes, less than 5% of the relative abundance comprised fungi (Figure S5D). Instead, unclassified Metazoa dominated the eukaryotic microbial communities, regardless of substrate type, plastic condition and age, with Chromalveolata, Stramenopiles, and unclassified Eukaryota abundant in all samples.

### 3.4 ASVs Indicative of Different Substrates and Biofilm Ages

Indicative species analyses were conducted on the amplicon data to determine which ASVs with more than 1% relative abundance were significantly different in their abundances based on substrate type and age (Figure S7). ASVs assigned to Acidobacteriota, Desulfobacterota, Firmicutes and Verrucomicrobiota were significantly indicative of plastisphere microbial communities. The genus *Halodesulfovibrio* was indicative of PET at 12 months, with a high abundance on PA at 9 months (Figure S7). LLDPE appeared to have the lowest number of indicative ASVs, with communities at 6 and 9 months having the least of two indicative ASVs, while 3‐ and 12‐month samples had the most at 11 and 10, respectively. All other plastics had at least double the number of indicators significantly associated with each substrate (59, 51, 65 and 70 indicator ASVs for OXO, PA, PET and PLA, respectively). Unknown ASVs accounted for approximately 60% of fungal indicators, with another 23% comprised of Chytridiomycota unassigned past phylum level (Figure S7B).

### 3.5 Broad‐Scale Functional Assignments of Metagenomic Data Based on Substrate Type, Age, Condition and Depth

**FIGURE 5:**
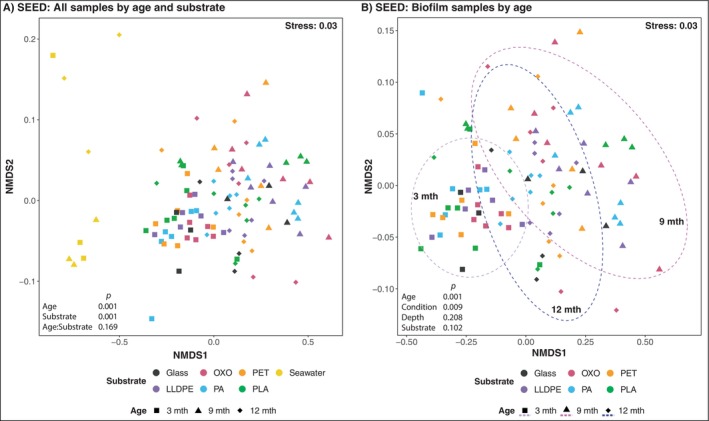
Non‐metric multi‐dimensional scaling (NMDS) ordination was used to visualise Bray–Curtis dissimilarity matrices of CSS‐normalised unassembled metagenomic SEED classification data from marine seawater and biofilm communities (i.e., on glass, LLDPE, OXO, PA, PET and PLA). Ellipses were drawn with the ‘stat_ellipse’ function of ggplot2, assuming a multivariate t‐distribution, and represent the variance observed among each (B) sampling age with confidence intervals of 95%.

The functional potential of biofilm communities significantly differed from those in seawater on a broad scale (PERMANOVA *p* = 0.001; Figure 5A), as supported by pairwise PERMANOVA (Figure S8A). Within biofilm communities, however, no significance was observed in the functional potential of microbial communities based on substrate (PERMANOVA *p* = 0.102; betadisper *p* = 0.765; Figures 5 and S8A,B), regardless of the inclusion of glass samples (PERMANOVA *p* = 0.115; betadisper *p* = 0.707). PERMANOVA revealed significant differences in functional gene classifications between non‐aged and aged communities (PERMANOVA *p* = 0.009; pairwise PERMANOVA *p* = 0.041). Similar to taxonomic classifications, age (i.e., length of sample immersion) was determined to be the primary driver of functional differences within all microbial communities, regardless of the inclusion of seawater samples and glass biofilms (PERMANOVA *p* = 0.001; Figures 5B and S8C,D), as observed by pairwise PERMANOVA (all comparisons *p* ≤ 0.011; Figure S8A). No significance was observed due to beta dispersion, regardless of whether seawater and glass sample data were included (betadisper *p* ≥ 0.418; all pairwise comparisons *p* ≥ 0.104; Figure S8B). Although greater beta dispersion among the data was evident with depth, PERMANOVA revealed no significant differences in functional classification among biofilm depths (PERMANOVA *p* = 0.235; all pairwise PERMANOVAs *p* ≥ 0.303; Figure S8B,D).

### 3.6 Functional Assignments Indicative of Different Substrates and Biofilm Ages

Of the SEED functions, seven were significantly indicative of certain substrates at different sampling times, two of which were associated with the plastisphere rather than seawater or glass‐associated communities: genes encoding the metabolism of amino acids and derivatives within LLDPE‐associated communities at 9 months, and those encoding carbohydrate metabolism were more abundant within PET‐associated communities at 12 months. Functional assignments related to metabolism dominated the microbiome data regardless of age, plastic condition, depth and substrate type (Figure S9), specifically amino acid and carbohydrate metabolism. Therefore, the overall functional potential of the communities within the marine environment appeared to be conserved.

### 3.7 Taxonomic Identification of ASVs Previously Reported as Being Related to Taxa With Plastic‐Degrading Potential and With More Than 0.1% Relative Abundance

**FIGURE 6:**
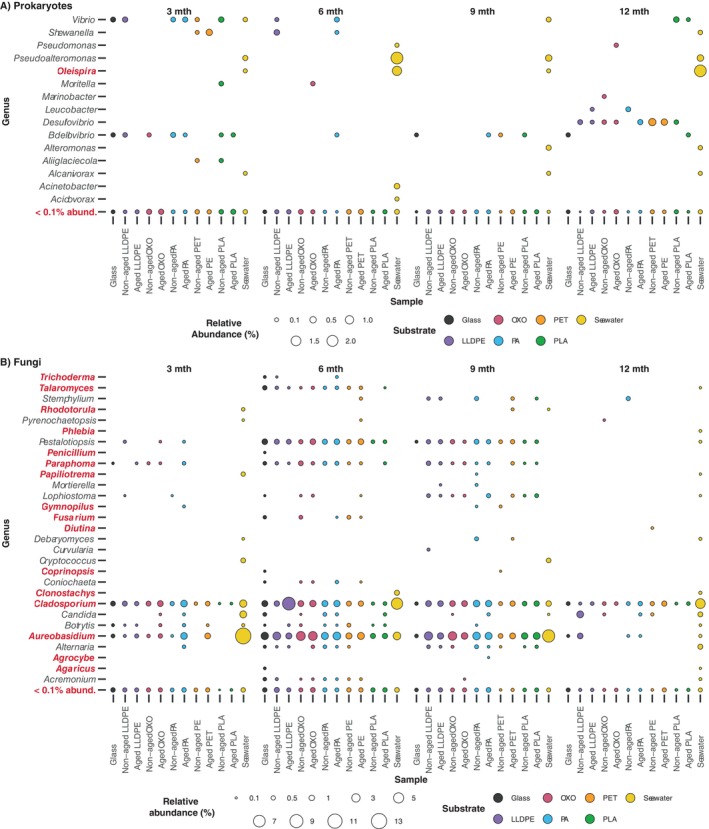
Relative abundance of (A) prokaryotic and (B) fungal genera related to previously reported plastic‐degrading microbes on glass, non‐aged and aged plastics (LLDPE, OXO, PA, PET and PLA) and in seawater over 12 months. Substrates were immersed in the marine environment of the Lighter Basin Marina, Tāmaki Makaurau‐Auckland Viaduct Harbour, Aotearoa‐New Zealand. Substrate type is shown by bubble colour, and bubble size represents relative abundance (%). Any genus with less than 0.1% relative abundance was grouped into a ‘< 0.1% rel abund.’ category. Potential plastic‐degrading genera identified at the species level are listed using bold text highlighted in red; the species names of genera with more than 0.1% relative abundance are listed in Table S5.

Genera closely related to previously reported plastic‐degrading microbes were identified on all substrates at all times (Figure 6). Of the genera found with more than 0.1% relative abundance per sample, *Bdellovibrio* was found exclusively in biofilms, while *Vibrio* was present in all biofilm samples except OXO, and was also abundant in the seawater. *Shewanella* was detected on PET, LLDPE, and PA at earlier biofilm ages and was only found in seawater on the final sampling occasion (12 months). As the biofilm matured, additional members of putative plastic‐degrading bacterial genera, such as *Desulfovibrio*, *Leucobacter* and *Marinobacter*, were detected. Most potential fungal plastic degraders were found at 6 and 9 months (Figure 6B).

Out of the 412 ASVs identified as indicator species (Figure S7A), only two prokaryotes were also reported to be plastic degraders, both of which belong to the phylum Proteobacteria; *Shewanella* was indicative of PET at 3 months, and *Pseudoalteromonas* was significant in seawater at 6 months. Three fungal ASV indicators were also previously reported to degrade plastics, all indicative of seawater samples (Figure S7B). The abundances of *Candida* and *Cryptococcus* were significantly indicative of seawater at 3 months, while *Clonostachys* was indicative of seawater sampled at 6 months.

### 3.8 Identification of Genes Conferring Putative Plastic Degradation Traits

**FIGURE 7:**
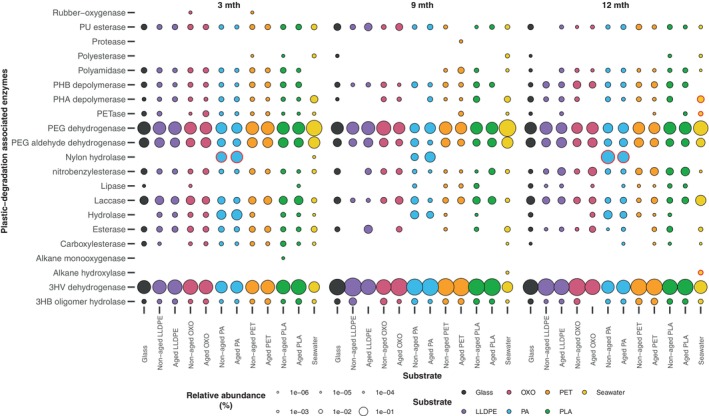
Bubble plot showing the relative abundance of genes previously identified to confer plastic degradation traits within microbial communities in seawater and biofilms (i.e., on glass and five plastic substrates), with five or more reads assigned based on a BlastP percentage identity of 70%. Enzymes associated with plastic degradation were obtained from PlasticDB (Gambarini et al. 2022). Substrates (i.e., glass, LLDPE, OXO, PA, PET and PLA) were deployed for up to 12 months in the marine environment of the Lighter Basin Marina, Tāmaki Makaurau‐Auckland Viaduct Harbour, Aotearoa‐New Zealand. Data bubbles relating to gene relative abundances identified as significantly associated (*p* > 0.05) with a specific substrate type and sampling point by IndVal analyses (Dufrêne and Legendre 1997) are outlined in red. The genera from which the different genes associated with plastic degradation have been attributed to are shown in Figure S10.

Most genes encoding enzymes associated with plastic degradation were identified in similar relative abundance in seawater, glass, and plastics, regardless of sampling age. The most prominent were related to polyethylene glycol (PEG) dehydrogenase, PEG aldehyde dehydrogenase and 3‐hydroxyvalerate (3 HV) dehydrogenase (Figure 7). Microbial communities on PA had sequences associated with nylon hydrolases, a low abundance of which was also found within sequences from seawater communities at 3 months.

While genes encoding purported plastic‐degrading enzymes were present across multiple substrates, some were present in plastic‐associated biofilms alone (Figure S10). Sequences associated with rubber‐oxygenase previously reported from *Steroidobacter cummioxidans* and *Rhizobacter gummiphilus* were observed in low abundance within non‐aged PET and non‐aged OXO metagenomes at 3 months, respectively. At 3 months, results associated with PETase (previously isolated from *Ketobacter* sp.) were observed exclusively in LLDPE, OXO and PA plastisphere metagenomes. Sequences predicted to encode a PHB depolymerase (previously isolated from *Azotobacter vinelandii* ) were observed solely in the plastisphere at three and 12 months. At 9 months, genes similar to a protease previously reported to be involved in PLA degradation were found in *Lederbergia lenta* within metagenomes associated with aged PET. Interestingly, while genes similar to those encoding hydrolases associated with plastic degradation were found on all substrates (Figure 7), a hydrolase‐related gene previously isolated from *Rhodopseudomonas palustris* was solely found in non‐aged PET samples. A gene similar to a hydrolase‐encoding gene found in *Paenarthrobacter ureafaciens* and reported to degrade nylon‐6 by‐products was observed at three and 12 months, exclusively within PA‐associated biofilms (Figure S10). Similarly, sequences associated with a nylon hydrolase from *Agromyces* sp. were observed at a higher relative abundance in PA samples than in all other substrates.

## 4 Discussion

The effects of plastic on marine microbial communities are poorly understood. Although it has previously been suggested that polymer type influences plastisphere community composition (Kirstein et al. 2018; McCormick et al. 2014; Zettler et al. 2013), we observed no significant difference in either overall community composition or metabolic potential due to plastic type or condition (i.e., whether the plastic was artificially aged before deployment). Instead, sample age was primarily determined to be the main factor shaping microbial communities' taxonomic and functional membership, regardless of substrate type (i.e., glass, LLDPE, OXO, PA, PET and PLA). Broadly, we found taxa previously associated with plastic degradation and plastic‐degrading genes at similar relative abundances on non‐aged and aged plastics, as well as associated with glass and seawater. Nevertheless, an increased abundance of nylon hydrolase genes was identified within our PA (nylon)‐associated communities.

While our amplicon and taxonomic metagenomic datasets were positively correlated, sequences from the latter were mostly unidentified at the genus level due to shorter query lengths (Tran and Phan 2020). One notable limitation in our study was the particularly weak correlations between fungal taxonomic assignments generated from the amplicon versus the shotgun metagenomic data. This may, in part, be caused by primer‐associated biases. While commonly used to assess fungal community diversity (Lear et al. 2018), the primer ITS4, for example, is known to be biased towards amplifying ascomycetes DNA (Bellemain et al. 2010), highlighting complexities for taxonomic interpretation of fungal amplicon sequence data. Additionally, many fungal amplicon reads could not be identified to the phylum level. Targeted metagenomic assembly and binning can improve classifications of presumptive plastic‐degrading taxa, noting that the abilities of many organisms and enzymes to degrade plastics remain unclear, or even controversial (Stepnov et al. 2024). However, the microbes associated with them are often rare, with genes conferring plastic degradation found in low abundance, making them less likely to be retained during assembly and binning. Our investigations were based on current knowledge of enzymes and taxa reported to degrade plastic, but many microbes in our datasets remain poorly characterised. These may represent taxa and enzymes with novel plastic degradation functionality that have yet to be formally described. Further phenotypic characterisation and metatranscriptomic analyses are needed to provide more insight into the active metabolism within the plastisphere and a more comprehensive perspective for the exploration of plastic degradation.

Weathering can adjust plastic polymers' surface chemistry, topography, and roughness (Andrady 2011), influencing polymer carbon bioavailability, microbial adhesion and subsequent community compositions (Donlan 2002). Although Laroche et al. (2023) demonstrated that UV ageing influenced diatom communities, it did not significantly affect prokaryotic and fungal diversity. Theobald et al. (2024) investigated the chemistry of our plastics, observing surface etching on aged PA, with sparse pinholes formed on aged PET and PLA. OXO changed the most with large surface cracks and etching observed; its mechanical properties deteriorated substantially. However, these changes did not lead to more diverse or distinct microbial communities than non‐aged plastics, suggesting UV ageing did not significantly influence microbial attachment or composition in the present study.

Prokaryotic communities were most diverse during the earliest sampling event at 3 months, regardless of plastic type or condition. This pattern reflects trends commonly observed for marine biofilm communities, including on plastics, where prokaryotic taxonomic richness peaks early during population growth, followed by a plateau (Bech et al. 2024) or decline in relative community richness or diversity. Indeed, marine biofilm communities undergo rapid temporal composition changes, with many taxa being highly intermittent or ephemeral (Pollet et al. 2018). As highlighted by Wallbank et al. (2022), it is likely that the earliest stages of colonisation represent the time when both plastic surface chemistry and polymer properties (e.g., the availability of contaminant monomers and oligomers for degradation) can exert the greatest selection pressure on the colonising community. As biofilms mature and become thicker, the extent of direct contact between the plastic's surface and most microbes within the biofilm may decrease, favouring the success of more ‘generalist’ taxa. Since previous studies have revealed plastic‐specific communities to be associated with younger biofilms (Erni‐Cassola et al. 2020; Harrison et al. 2014; Wallbank et al. 2022), this indicates a possible need for finer‐resolution temporal sampling to distinguish community variation due to substrate factors. In the present study, however, the highest relative abundances of putative plastic‐degrading taxa appeared after six or more months of development in seawater, possibly reflecting the greater abundance of organisms capable of degrading more complex carbon substrates that may be present in older biofilms, including increased volumes of chitin and cellulose polymers.

Interestingly, we found few differences in community composition comparing the microorganisms on different plastic polymers or on the glass control treatments. Since glass is chemically inert and microorganisms cannot use glass as a carbon or energy source, this reinforces that the organisms present on the different plastics could not gain enough carbon from the plastics to cause substantial changes in community composition or functioning among treatments. Consequently, our study contributes to a growing body of evidence that these communities may primarily reflect general biofilm succession that may occur on any submerged inert surface, rather than the ‘plastisphere’ representing a unique plastic‐specific niche (Coons et al. 2021). Thus, instead of housing distinct novel microbiomes, perhaps a more important feature of marine plastics is their provision of a huge surface area for the microbial colonisation of solid surfaces in the upper water column (i.e., noting ~32,000 kt of buoyant plastic items are predicted to be present in the global marine environment; Kaandorp et al. 2023). As shown in this and other studies, microorganisms with the potential to degrade plastics are characteristically scarce within the plastisphere, and even where present, the active degradation of plastic is rarely confirmed. In contrast to glass substrates, previous studies have determined that natural organic substrates such as wood do host distinct microbial communities compared to plastics (Kettner et al. 2019; Kettner et al. 2017), as taxa with the ability to degrade those substrates gain metabolic advantage, even after relatively short periods of immersion in the marine environment (e.g., 4 weeks, Silva et al. 2023). We recommend that future studies include hydrophilic natural organic control treatments, such as wood, to better quantify changes in community composition related to substrate degradation, where it may occur.

As with the microbial community composition, sampling age was the main factor driving diversity among the functional potential of communities on plastic and glass, with no significance attributed to plastic type, supporting the findings of Maday et al. (2024). Plastic condition played a role in the diversity of functional gene assignments in our study, with aged plastic communities possessing a higher diversity of functional genes. However, overall biological pathways were conserved among all microbial communities; they appeared to retain a core general metagenome, as was previously observed by Philippot et al. (2021).

Biofilms forming on plastics may harbour microbes that can degrade plastic polymers, offering novel opportunities for bioremediation. Taxa associated with plastic degradation have been identified within the plastisphere (Agostini et al. 2021; Wallbank et al. 2022), and studies have found the enrichment of genes potentially conferring plastic degradation (Bryant et al. 2016; Debroas et al. 2017; Pinnell and Turner 2019; Zhou et al. 2023). Our study identified such taxa and genes on plastics, glass, and seawater, albeit at very low relative abundances. The sole purported plastic degrader identified with a relative abundance indicative of the substrate it resided on was *Shewanella* on PET samples at 3 months. This bacterium has previously been reported to degrade polycaprolactone (PCL) and polyhydroxyalkanoate (PHA), but not PET (Sekiguchi et al. 2011; Sung et al. 2016; Suzuki et al. 2017). Since evidence for the microbial degradation of most plastics is lacking (Lear et al. 2022), identifying the mechanisms by which degradation occurs is critical for determining whether the taxa found in environmental samples have the functional potential to degrade plastic, if at all. Further confirmation of plastic degradation by microbial enzymes is necessary since increases in the activity of these enzymes may merely be related to their interaction with natural substrates, such as lignin and chitin.

Whilst genes conferring plastic degradation traits have been found in fungi (Gambarini et al. 2021) and Archaea (Perez‐Garcia et al. 2023), our samples contained only genes previously reported within Bacteria. Within our samples, *Pseudomonas* was the only genus identified as closely related to plastic‐degrading microbes and present on all plastic types and glass. This genus can produce biosurfactants, aiding in the biodegradation of hydrophobic pollutants (Hu et al. 2023). Genes identified from *Pseudomonas* within our samples include those reported to encode polyesterase, PHA depolymerase, lipase, hydrolase, and alkane hydroxylase. Plastics these enzymes can reportedly degrade include LDPE (Montazer et al. 2019; Skariyachan et al. 2017), PLA (Bubpachat et al. 2018) and PET (Bollinger et al. 2020), suggesting *Pseudomonas* sp. may be able to degrade multiple plastic polymers or their degradation products; yet, these attributes remain to be tested with appropriate enzyme assays.

Within the plastisphere, most genes associated with plastic degradation were found in similar abundances on aged and non‐aged plastics alike, regardless of the plastic type. Such genes are often involved in more general metabolic pathways. For example, genes in *Paracoccus denitrificans* (Lu et al. 2014) encoding dehydrogenases may degrade plastics derived from biomass such as PHA and polyhydroxybutyrate (PHB). Laccases can degrade the highly abundant aromatic polymer lignin (Janusz et al. 2017), with those found in *Psychrobacter* sp. reportedly degrading PE (Zhang et al. 2022). They, therefore, may not serve the purpose of plastic degradation, but instead, perform a more general function within microbial communities in the marine environment. Interestingly, genes associated with nylon degradation, that is, hydrolase and nylon hydrolases, were enriched and significantly indicative of our PA (nylon) communities. This was consistent with the findings of Maday et al. (2024), who found the same genes within metagenomes of aged PA‐associated communities in a wastewater treatment plant in Ōtautahi‐Christchurch, A‐NZ. Theobald et al. (2024) found evidence of changes in the surface morphology of non‐aged PA over time that were indicative of biological erosion, suggesting the microbial communities on PA may contribute to the physical and mechanical wear of plastics in the marine environment. Yasuhira et al. (2007) discovered genes encoding nylon hydrolase in *Agromyces* sp. and *Kocuria* sp., while Kinoshita et al. (1975) discovered genes encoding hydrolases in *Paenarthrobacter ureafaciens*, all of which were significantly associated with our PA communities. While this highlights the potential of our PA biofilms to possess/retain genes conferring nylon degradation traits, the enzymes they encode are confirmed only to degrade nylon‐6 by‐products (i.e., dimers and oligomers). Further, the relative abundance of these genes was low compared to other functional genes and the associated taxa were not identified within our communities. Further research is needed to determine the origin and activity of these genes and to confirm their potential for degrading nylon polymers, their degradation products, or contaminants.

## 5 Conclusion

With the continual accumulation and longevity of plastic in our oceans, research into the long‐term impacts of plastic pollution on marine microbial communities is essential to determine the risks posed to our oceanic microbiota and the plastic‐degrading potential they may harbor. Our study provides new insights into the effects of five abundant plastic polymers, both aged and non‐aged, on the diversity and composition of prokaryotic and fungal communities, as well as their functional potential. We found generalist communities on all plastics, with conserved functional traits. Although plastic‐specific communities were not identified, we found genetic attributes linked to potential plastic‐degrading enzymes. Overall, our study enhances our understanding of the communities colonising plastic in the marine environment, including their presumed plastic‐degrading potential.

## Author Contributions

Conceptualisation: J.A.W., O.P., J.M.K., L.W., G.L. Data curation: J.A.W., J.M.K. Formal analysis: J.A.W., V.G. Funding acquisition: O.P. Investigation: J.A.W., J.M.K., O.P., L.W., D.A.S., M.B., B.T., V.G., G.L. Methodology: J.A.W., D.A.S., M.B., B.T., V.G. Project administration: O.P., J.A.W. resources, O.P., L.W., J.M.K. Supervision: G.L., O.P., J.M.K., L.W. Validation: J.A.W., V.G., D.A.S. Visualisation: J.A.W. writing – original draft: J.A.W., G.L., O.P., L.W., J.M.K. writing – review and editing: J.A.W., J.M.K., O.P., L.W., D.A.S., M.B., B.T., V.G., G.L.

## Funding

This work was supported by the Ministry of Business, Innovation and Employment (C03X1802).

## Conflicts of Interest

The authors declare no conflicts of interest.

## Supporting information


**Data S1:** Supporting Information.

## Data Availability

The datasets presented in this study can be found in the Sequence Read Archive (SRA) of the National Centre for Biotechnology Information (NCBI) under the BioProject: PRJNA1163745 (Reviewer link: https://dataview.ncbi.nlm.nih.gov/object/PRJNA1163745?reviewer=79qb7789ic6tslg4e1tjqmn5iv).
