# Supplementary material for: Plastic Type and Condition Have Minimal Impact on Associated Marine Biofilm Communities
Source: Environ Microbiol. 2025 Dec 12;27(12):e70214. doi: 10.1111/1462-2920.70214 (PMC12701327; doi:10.1111/1462-2920.70214)
Supplement: Supplementary file 1 — Data S1: Supporting Information. [file EMI-27-e70214-s001.docx]

**Supplementary Methods**

*Sample processing*

As detailed by Wallbank *et al.* (2022), the deployment structure was entirely removed from the water on each sampling occasion. Pre-labelled individual sterile sample bags (Cat No. 14955189; Thermo Fisher Scientific, Waltham, MA, USA) were placed carefully around each paddle before the integrated arm was excised from the structure using sterile pipe cutters. Nitrile-free gloves were used throughout sampling, being replaced between substrate types. At each sampling time, three field sampling controls were also collected by opening empty sample bags on site. In parallel, three 2 L seawater samples were collected at each sampling time from the exact location and approximate depth of each deployment structure to determine ambient seawater microbial community compositions. A stainless-steel water collection bucket and 2 L Schott Duran® glass bottles were sterilised by acid-washing and autoclaving before use. It was rinsed thrice with seawater in the field before sampling. All samples were placed on ice for transportation back to the laboratory for processing within 2 hours of sampling.

Biomass was removed from both sides of the substrates using individual sterile flat-edged razor blades and placed into pre-labelled sterile 50 ml centrifuge tubes. The scraped plastic or glass was returned to the sampling bag with 30 ml ice-cold, sterile Tris-EDTA buffer (TE; Tris 10 M, EDTA 1mM, pH 8.0). To recover biomass firmly attached to the plastic or glass, sample bags were placed in an ice ultrasonic water bath (Bandelin Sonorex RK 100H; Sigma-Aldrich, St. Louis, MO, USA) and sonicated at 35 kHz for 2 min. Following sonication, the sonicate solution and the recovered biomass from the same sample were homogenised together with two 9.5 mm sterile stainless-steel beads for 2 min at 1,200 rpm (GenoGrinder^®^ 2010; Spex SamplePrep, Metuchen, NJ, USA). Samples were then aliquoted into three 5 ml centrifuge tubes and centrifuged at 4,500 x *g* for 10 min at 4°C. Supernatants were gently discarded by decanting, and the process was repeated until no biomass remained. Centrifugation for an additional 1 min then occurred, followed by removing the remaining supernatant by pipette. Seawater was filtered (one litre per filter) through 0.2 µm filter membranes (Supor® 200, Whatman) using a vacuum pump. Membranes were placed in individual, sterile 5 ml centrifuge tubes, and all samples were stored at -80°C until required.

DNA was extracted from up to 250 mg of biomass for each sample. To assist with sample handling, 100 µl UltraPure DNase/RNase-free distilled water (Cat No. 10977015; Invitrogen, Thermo Fisher Scientific, Waltham, MA, USA) was used to resuspend sample masses <100 mg and the entire volume was used for extraction. All steps were performed according to the manufacturer’s instructions, except for the mechanical lysis step, which used a TissueLyser (Cat No. 85300; Qiagen, Hilden, Germany) for 2 min at 30 Hz. For the seawater samples, sterile tweezers were used to place filters directly into the PowerSoil bead tubes. Extraction kit blanks were completed at the start and end of every round of extraction.

*PCR and DNA amplicon sequencing*

Amplification of the small-subunit (16S) of the hypervariable V4 region of prokaryotic ribosomal RNA (rRNA) genes was performed by polymerase chain reactions (PCRs) using the universal amplicon primer pair 515F and 806R (see Table S2). Fungal DNA was also amplified, targeting the fungal internal transcribed spacer 2 (ITS2) region of the nuclear ribosomal gene using the primer pair fITS7 and ITS4 (Table S2). Illumina adapter sequences were included in all primers for amplicon sequencing (Kozich *et al.*, 2013). A total reaction volume of 25 µl was used for all PCRs, including controls. It was comprised of 0.25 µl MTP™ Taq DNA Polymerase, 2.5 µl 10X MTP™ Taq buffer (Invitrogen, Thermo Fisher Scientific, Waltham, MA, USA), 0.75 µl of each primer (10 µM), 1 µl template DNA and 10 µl UltraPure DNase/RNase-free distilled water (Cat No. 10977015; Invitrogen, Thermo Fisher Scientific, Waltham, MA, USA). Amplicon sizes were verified by gel electrophoresis. PCR products (4 µl) were run on a 0.8% (w/v) agarose gel. SYBR SAFE DNA Gel Stain (Invitrogen, Thermo Fisher Scientific, Waltham, MA, USA) was added for DNA visualisation under UV light using a GelDoc imaging system. Quantification of PCR products was performed using a Qubit double-stranded DNA (dsDNA) High Sensitivity Assay kit (Invitrogen, Thermo Fisher Scientific, Waltham, MA, USA) before purification via a DNA Clean and Concentrator-5 kit (Zymo Research, Irvine, CA, USA). Purified PCR products were eluted in 15 µl DNA elution buffer, according to the manufacturer’s instructions, and diluted to 3 ng/µl using UltraPure DNase/RNase-free distilled water. A negative control of UltraPure DNase/RNase-free distilled water was included on each run, alongside amplified mock microbial standard controls (ZymoBIOMICS Microbial Community and DNA standards) for each target amplicon to determine bias in amplification and sequencing (Zymo Research, Irvine, CA, USA). Samples were sent to the Auckland Genomics Facility (The University of Auckland, New Zealand) for further library preparation and sequencing. A unique combination of Nextera XT dual indices (Illumina Inc., USA) was attached to the DNA from each sample for sample multiplexing before sequencing on an Illumina MiSeq instrument using two‐by‐300‐bp V3 chemistry.

*Processing and analysis of DNA amplicon sequence data*

The sequencing provider demultiplexed sequences before any processing or analysis. Sequence adaptors were removed from all amplicons using Trimmomatic (version 0.39; Bolger *et al.*, 2014), adopting the parameters ILLUMINACLIP:2:40:15. Cutadapt (Martin, 2011) was used to remove fungal primers due to variations in the length of the ITS2 region before subsequent processing using the DADA2 package (version 1.26.0; Callahan *et al.*, 2016) in R (version 4.2.1; R Core Team, 2023). Prokaryotic primers were removed during the filtering and trimming step of the DADA2 package, and reads were then processed following the package instructions. Briefly, sequence read quality was checked; reads were filtered using a maximum estimated error (“maxEE”) of 2 bp per 100 bp. Sequence error rates were estimated by pooling samples to achieve convergence of the parametric error model. Samples were dereplicated and pooled for amplicon sequence variant (ASV) inference to reduce low sampling bias. Forward and reverse reads were merged, chimeric sequences were identified and removed, and an ASV table was produced. Compared to other approaches with more traditional clustering, DADA2 resolves sequences at a single nucleotide level, allowing biological variants to be identified more accurately; ASV cluster inference was defined at 100% similarity. The naïve Bayesian classifier method was implemented to assign prokaryotic taxonomy using the 16S rRNA gene SILVA reference database (version 138.1; McLaren & Callahan, 2021) and the UNITE ITS reference database (general FASTA release version 9.0 (18.07.2023); Abarenkov *et al.*, 2023) for fungal taxonomic classification. ASVs that remained unassigned as Archaea, Bacteria or Fungi at the kingdom level were removed, as were those classified as originating from mitochondria and chloroplasts.

Raw metagenomic sequence reads were quality-checked using FastQC (version 0.11.9; Andrews, 2010) before filtering, and adaptor-trimming was performed by Trimmomatic using TruSeq3-PE-2.fa to remove universal Illumina adaptors added to the sequences during library preparations. Sequence reads with a single read score below 15, paired read score below 40 and >2 bp mismatch were discarded, and leading and trailing ends with a quality score below three were removed. The quality of trimmed metagenomic reads was rechecked using FastQC before high-quality reads were concatenated based on individual samples to combine multiple lanes and sequence runs. To assign taxonomy, partial archaeal, bacterial, fungal, and eukaryotic small subunit (SSU) and large subunit (LSU) rRNA sequences were trimmed and merged. Taxonomic classifications against the SILVA database were obtained using Metaxa2 (version 2.2.3; Bengtsson-Palme *et al.*, 2015). Singletons and sequences unidentified to Archaea, Bacteria and Eukarya at the kingdom level were removed.

*Quantitative analyses*

Environmental data were analysed using analysis of variance (ANOVA) using the “aov” function of the R ‘stats’ package (version 4.4.1; R Core Team, 2023). Standard linear mixed modelling (LMM) was performed using the “lmer” function of the ‘lme4’ R package (version 1.1-35.6; Bates *et al.*, 2015) to determine the significance between illuminance at each depth while accounting for sampling age (1|Age), with the significance of the models assessed via likelihood ratio testing using “confint” function of ‘stats’.

For alpha diversity analyses only, ASV tables were rarefied using set.seed(123) via the “rarefy_even_depth” function of the ‘phyloseq’ R package (version 1.42.0; McMurdie & Holmes, 2013). To investigate taxonomic evenness and richness within and among samples, Chao1, Shannon and inverse Simpson statistics were calculated using the “estimate_richness” function of the ‘phyloseq’ package. Post-hoc pairwise comparisons were performed using the ‘pairwise.wilcox.test’ function from ‘stats’, and medians were calculated to determine the direction of the significance.

For all other analyses, cumulative sum scaling (CSS) normalisation was performed on non-rarefied data using the ‘metagenomeSeq’ R package (version 1.43.0; Paulson *et al.*, 2013) to correct for differences in sequencing depth and ensure comparable diversity. To determine whether age, condition, depth and substrate type statistically influenced microbial community compositions and functional potential, the “adonis2” function of the ‘vegan’ R package (version 2.6.4; Oksanen *et al.*, 2020) was used to perform permutational multivariate analysis of variance (PERMANOVA) with 999 permutations on Bray-Curtis dissimilarity matrices. To assess statistical significance (*P* < 0.05) between microbial community functional potentials and compositions, separately, based on substrate type, biofilm age, plastic condition (i.e. whether the plastic was UV ‘aged’ and ‘non-aged’) and sampling depth, pairwise PERMANOVAs were conducted using the ‘vegan’ wrapper “pairwise.adonis” function of ‘pairwiseAdonis’ (version 0.4.1; Martinez Arbizu, 2020). In contrast, the function “betadisper” was used to quantify the homogeneity of multivariate variance. Seawater samples were assigned as ‘Seawater’ as they were collected across the three depths due to variation between depths being reduced by the lateral and vertical movement of water from ocean currents. Non-metric multidimensional scaling (NMDS) was used for clustering visualisation with the ‘ggplot2’ R package (version 3.4.3; Wickham, 2016). A multivariate t-distribution was assumed when ellipses were added onto NMDS plots using the “stat_ellipse” function of ‘ggplot2’. Relative abundances of phyla were plotted to assess overall taxonomic differences within the microbial communities on different substrate types over time. Samples were pooled at each sampling time to investigate microbial community compositions based on substrate type and plastic condition. Stacked bar plots showing the relative abundances of the five most abundant genera present per substrate were created separately for comparison. Indicative value (IndVal) analyses with 999 permutations were performed on prokaryotic and fungal ASVs with more than 1% relative abundance per sample for each sampling time and substrate type to determine which significantly influenced the differences in microbial community compositions (Dufrêne & Legendre, 1997). The default parameters of MAFFT were used to align sequences identified as significant (version 7.505; Katoh & Standley, 2013) and constructed into trees using FastTree (Price *et al.*, 2010). Trees were then visualised and annotated using the web-based tool iTOL (version 6; Letunic & Bork, 2024). CSS-normalised metagenomic read counts were combined based on KEGG Level 2 classifications, and a heatmap was plotted using ‘ggplot2’.

Amplicon taxonomy tables were used to identify microbes closely related to those in PlasticDB, a database containing previously reported plastic-degrading microorganisms and enzymes (Gambarini *et al.*, 2022). ASVs were identified at the genus level, with few identified to species level.

**Supplementary Material**

**Table S1 | Constitution of substrates used in this study, as previously reported by Theobald *et al.* (2024), Laroche *et al.* (2023) and Wallbank *et al.* (2025).** Known additives were added by Scion, Rotorua, NZ.

| Substrate | Referred to in this paper as | Base polymer^1,2^ | Known additives | Additive role |
| --- | --- | --- | --- | --- |
| Glass | Glass | Low iron soda-lime float  glass | None | None |
| Linear low-density polyethylene | LLDPE | Innoplus LL7410D1 | 0.25% Irganox B215 (33% Irganox® 1010 (CAS 6683-19-8) and 67% Irgafos® 168 (CAS 31570-04-4)^3^ | Processing and long-term thermal stabiliser |
| Oxo-linear low-density polyethylene | OXO | Innoplus LL7410D1 | 0.2% manganese stearate (CAS3353-05-7) | Oxo-degradable additive |
| Nylon-6 | PA | Ultramid B3S | Talc | Bulking/filler agent |
|  |  |  | 0.5% nylostab S-EED (CAS 422774-15-2) | Stabiliser |
| Polyethylene terephthalate | PET | PAPETCOOL IV0.80 (standard bottle grade) | 0.3% Tinuvin 234 (CAS 70321-86-7) | UV absorbent |
| Polylactic acid | PLA | Ingeo 3052D | < 0.2% ethylene bis(stearamide) (CAS 110-30-5) | Lubricant additive |

^1^Inorganic content of PA, PET and PLA polymers was quantified as < 0.1% of the original mass of the samples (below the detection limit) by thermogravimetric testing.

^2^Inorganic content of LLDPE base polymers was quantified as < 0.5% of the original mass of the samples (just above the detection limit) by thermogravimetric testing.

^3^0.13 wt% AO-1076, an antioxidant stabiliser, was detected in virgin polyethylene resin (Bridson *et al.*, 2023).

**Table S2 | PCR primers and conditions for targeting the hypervariable V4 region of the prokaryotic small-subunit of ribosomal RNA (16S rRNA) gene and the fungal internal transcribed spacer 2 (ITS2) region of the nuclear ribosomal gene.** Underlined sequences represent the Illumina Nextera adaptor overhang required for sample indexing (Kozich *et al.*, 2013).

| Targeted taxa | Gene fragment amplified^1^ | Primer name | Sequence (5’ - 3’) | Annealing temp (°C) | Primer reference |
| --- | --- | --- | --- | --- | --- |
| Prokaryotes | 16S rRNA  (V4 region) | 515F_adpt | TCG TCG GCA GCG TCA GAT GTG TAT AAG AGA CAG GTG YCA GCM GCC GCG GTA A | 50^2^ | Parada *et al.* (2016) |
|  |  | 806R_adpt | GTC TCG TGG GCT CGG AGA TGT GTA TAA GAG ACA GGG ACT ACN VGG GTW TCT AAT |  | Apprill *et al.* (2015) |
| Fungi | ITS  (ITS2 region) | fITS7_adpt | TCG TCG GCA GCG TCA GAT GTG TAT AAG AGA CAG GTG ART CAT CGA ATC TTT G | 52^3^ | Ihrmark *et al.* (2012) |
|  |  | ITS4_adpt | GTC TCG TGG GCT CGG AGA TGT GTA TAA GAG ACA GTC CTC CGC TTA TTG ATA TGC |  |  |

^1^Post-PCR purifications were performed using a DNA clean and concentrator-5 kit (Zymo Research, Irvine, CA, USA). All amplicons were sequenced on an Illumina MiSeq instrument by the Auckland Genomics Facility (The University of Auckland, New Zealand).

^2^Initial denaturation (94 °C for 3 min); 30 cycles of denaturation (94°C for 45 sec), annealing (50°C for 1 min), extension (72°C for 90 sec); final extension (72°C for 10 min).

^3^Initial denaturation (94 °C for 5 min); 30 cycles of denaturation (94°C for 30 sec), annealing (52°C for 30 sec), extension (72°C for 45 sec); final extension (72°C for 10 min).

**Table S3 | Averaged daily light intensity (lux) and temperature (°C) at each depth of the structure deployed in the Lighter Basin Marina, Tāmaki Makaurau-Auckland Viaduct Harbour, Aotearoa-New Zealand.** Dataloggers were positioned at 20, 40 and 60 cm below the water's surface (referred to as Top, Middle and Bottom, respectively), and readings were averaged for the three days post-sampling.

| Time | Datalogger position | Temperature  (°C) | Lux | Average minutes of light per day |
| --- | --- | --- | --- | --- |
|  |  |  |  |  |
| Deployment | Top | 21.61 | 3486 | 775 |
| (March) | Middle | 21.60 | 4279 | 765 |
|  | Bottom | 21.50 | 3507 | 765 |
| 3 months | Top | 14.28 | 2026 | 630 |
| (June) | Middle | 14.32 | 864 | 620 |
|  | Bottom | 14.59 | 641 | 615 |
| 6 months | Top | 14.81 | 12575 | 765 |
| (September) | Middle | 14.62 | 4506 | 765 |
|  | Bottom | 14.41 | 6236 | 760 |
| 9 months | Top | 21.95 | 16587 | 875 |
| (December) | Middle | 21.90 | 11918 | 930 |
|  | Bottom | 21.67 | 8442 | 930 |
| 12 months* | Top | 22.39 | 8506 | 800 |
| (March) | Middle | 22.34 | 3091 | 800 |
|  | Bottom | 22.09 | 3851 | 805 |

*As the structure was removed entirely after the final sampling point, measurements were taken for three days before sampling.

**Figure S1 | Relative abundance (%) of genera identified in (A) bacterial and (B) fungal mock microbial community controls.** MOCK-EXT was omitted for fungi as no reads were retained for the fungal mock extraction following DADA2 processing. MOCK-STANDARD represents the expected theoretical compositions.

**Figure S2 | Rarefaction curves of (A) prokaryotic and (C) fungal communities on glass, plastics and seawater in the marine environment of the Lighter Basin Marina, Tāmaki Makaurau-Auckland Viaduct Harbour, Aotearoa-New Zealand.** Prokaryotic and fungal amplicon reads were rarefied to 1438 and 2260, respectively. Alpha diversity indices (Chao1, Shannon and inverse Simpson) of rarefied (B) prokaryotic and (D) fungal communities were investigated. The box shows the 25^th^, 50^th^ and 75^th^ percentiles, with dots representing outliers. Standard error is shown by error bars, with lowercase letters representing statistical significance at each age by each substrate (Tukey’s HSD, *P* < 0.05).

**Table S4 | Analysis of variance (ANOVA) investigating Chao1, Shannon and Inverse Simpson factors such as age, depth, substrate and their interaction terms of all samples, biofilm samples and plastic-associated samples used in this study**. Only amplicon data are included. Significant *P* values (*P* < 0.05) are highlighted in bold.

| **Terms** | **Prokaryotes** | | | | | **Fungi** | | | | | |
| --- | --- | --- | --- | --- | --- | --- | --- | --- | --- | --- | --- |
|  | Chao1 | Shannon | | Inverse Simpson | | Chao1 | | Shannon | | Inverse Simpson | |
| All samples: |  |  | |  | |  | |  | |  | |
| Age | **0.001** | | **0.001** | | **0.001** | | **0.001** | | **0.001** | | **0.001** |
| Substrate | **0.001** | | **0.001** | | **0.001** | | **0.001** | | **0.001** | | **0.019** |
| Condition | 0.243 | | 0.972 | | 0.148 | | 0.055 | | 0.214 | | 0.370 |
| Age*Substrate | **0.001** | | **0.001** | | **0.001** | | **0.017** | | **0.005** | | **0.001** |
| Age*Condition | **0.009** | | 0.498 | | **0.018** | | 0.844 | | 0.565 | | 0.292 |
| Substrate*Condition | 0.142 | | 0.339 | | 0.541 | | 0.444 | | 0.319 | | 0.058 |
| Age*Substrate*Condition | 0.690 | | 0.316 | | 0.569 | | 0.987 | | 0.968 | | 0.121 |
| Seawater samples removed: | |  | |  | |  | |  | |  | |
| Age | **0.001** | | **0.001** | | **0.001** | | **0.001** | | **0.001** | | **0.001** |
| Depth | **0.002** | | **0.031** | | **0.033** | | **0.002** | | 0.187 | | 0.532 |
| Substrate | **0.007** | | 0.078 | | **0.036** | | **0.001** | | **0.001** | | **0.023** |
| Age*Depth | 0.145 | | 0.233 | | 0.174 | | **0.001** | | **0.026** | | 0.115 |
| Age*Substrate | 0.080 | | 0.451 | | 0.125 | | **0.001** | | **0.021** | | **0.001** |
| Depth*Substrate | 0.534 | | 0.439 | | 0.093 | | **0.002** | | 0.058 | | 0.252 |
| Age*Depth*Substrate | 0.927 | | 0.798 | | 0.403 | | 0.600 | | 0.329 | | 0.625 |
| Seawater and glass samples removed: | | | |  | |  | |  | |  | |
| Age | **0.001** | | **0.001** | | **0.001** | | **0.001** | | **0.001** | | **0.001** |
| Depth | **0.007** | | **0.027** | | **0.034** | | 0.057 | | 0.070 | | 0.477 |
| Substrate | 0.085 | | 0.372 | | 0.270 | | **0.001** | | **0.002** | | 0.253 |
| Age*Depth | 0.181 | | 0.184 | | 0.194 | | **0.002** | | **0.049** | | 0.243 |
| Age*Substrate | 0.055 | | 0.392 | | 0.057 | | 0.074 | | 0.480 | | 0.461 |
| Depth*Substrate | 0.601 | | 0.315 | | 0.069 | | 0.253 | | 0.106 | | 0.315 |
| Age*Depth*Substrate | 0.894 | | 0.619 | | 0.435 | | 0.733 | | 0.347 | | 0.575 |

**Figure S3 | (A and B) Pairwise PERMANOVA, (C and D) betadisper comparisons and (E and F) non-metric multidimensional scaling (NMDS) plots on Bray-Curtis dissimilarity matrices from amplicon DNA sequences.** Permuted betadisper *P*-values are shown above the diagonal, and observed betadisper values are below. Colour and asterisks represent significance: yellow – *P* < 0.05 (*), orange – *P* < 0.01 (**) and red – *P* < 0.001 (***). Each point on the plots represents individual (E) prokaryotic and (F) fungal samples shaped by biofilm age and coloured by substrate. A multivariate t-distribution was assumed when drawing ellipses using the “stat_ellipse” function of ggplot2. Ellipses represent the variance observed among each sampling age, with confidence intervals of 95%. Seawater data were excluded from the multivariate plots.

**Figure S4 | Bray-Curtis dissimilarity matrix of taxonomic assignments of unassembled metagenomic reads from microbial communities analysed by (A) pairwise PERMANOVA and (B) pairwise betadisper comparisons, visualised by (C, D, E and F) non-metric multidimensional scaling (NMDS) plots.** Samples were obtained from seawater and biofilms on glass and plastic (i.e. LLDPE, OXO, PA, PET and PLA) deployed for up to 12 months in the marine environment of the Lighter Basin Marina, Tāmaki Makaurau-Auckland Viaduct Harbour, Aotearoa-New Zealand. (A and B) Colour and asterisks represent significance: yellow – *P* < 0.05 (*), orange – *P* < 0.01 (**) and red – *P* < 0.001 (***). (B) Permuted betadisper *P*-values are shown above the diagonal, and observed betadisper values are below. (D, E and F) Ellipses were drawn with the “stat_ellipse” function of ggplot2, assuming a multivariate t-distribution. Confidence intervals of 95% were used to represent the variance observed among each (D) sampling age, (E) plastic condition and F) sampling depth.

**Figure S5 | Proportion of SSU and LSU rRNA fragments identified in metagenomic data and assigned to the (A) archaeal (0.04%), bacterial (21.60%) and eukaryotic (78.36%) domains of life.** Samples were taken of seawater and from biofilms growing on plastic and glass deployed for up to 12 months in the marine environment of the Lighter Basin Marina, Tāmaki Makaurau-Auckland Viaduct Harbour, Aotearoa-New Zealand. CSS-normalised relative abundance (%) of (B) bacterial, (C) archaeal and (D) eukaryotic phyla within biofilms growing on glass, plastics and ambient seawater.

**Figure S6 | Relative abundance (%) of (A) prokaryotic and (B) fungal phyla from taxonomic analyses of amplicon data from biofilm samples taken from glass, plastics and seawater in the marine environment of the Lighter Basin Marina, Tāmaki Makaurau-Auckland Viaduct Harbour, Aotearoa-New Zealand.**

| **Kingdom** | **Genus** | **Species** |
| --- | --- | --- |
| Bacteria | Oleispira | antarctica |
| Fungi | Agaricus | bisporus |
|  |  | subrufescens |
|  | Agrocybe | praecox |
|  | Aureobasidium | pullulans |
|  | Cladosporium | sphaerospermum |
|  |  | tenuissimum |
|  | Clonostachys | buxi |
|  |  | rosea |
|  | Coprinopsis | lagopus |
|  | Diutina | rugosa |
|  | Fusarium | equiseti |
|  |  | graminearum |
|  |  | oxysporum |
|  |  | tricinctum |
|  | Gymnopilus | junonius |
|  | Papiliotrema | laurentii |
|  | Paraphoma | radicina |
|  | Penicillium | expansum |
|  | Phlebia | radiata |
|  | Rhodotorula | mucilaginosa |
|  |  | sphaerocarpa |
|  | Talaromyces | minioluteus |
|  |  | variabilis |
|  | Trichoderma | hamatum |
|  |  | harzianum |
|  |  | virens |
|  |  | viride |

**Table S5 | Plastic-degrading genera identified with more than 0.1% relative abundance within study samples (highlighted in red in Figure 6) using PlasticDB (Gambarini *et al.*, 2022) and their associated species epithets.**

**Figure S7 | (A) Bacterial and (B) fungal ASVs with a relative abundance of >1% displayed in phylogenetic trees that were indicative of substrate type (i.e. five plastics, glass or ambient seawater) after incubation of up to 12 months in the marine environment of the Lighter Basin Marina, Tāmaki Makaurau-Auckland Viaduct Harbour, Aotearoa-New Zealand.** Samples were taken at months 3, 6, 9 and 12. Monte Carlo tests using 999 permutations were used to assess indicator species (IndVal) analyses, with significance determined as *P* < 0.05. Shapes between branches and leaves represent biofilm age, with its colour showing substrate type. The colour of the leaves represents phyla, with blue bars representing the relative abundance of each ASV within the sample it is indicative of. The teal text highlights the genera related to those previously reported as degraders. Interactive versions of these trees are available: ‘Long-term deployment IndVal trees’ - <https://itol.embl.de/shared/jwal670>.

**Figure S8 | Bray-Curtis dissimilarity matrix of SEED functional classifications of CSS-normalised unassembled metagenomic reads within microbial communities analysed by (A) pairwise PERMANOVA and (B) betadisper comparisons visualised by (C and D) non-metric multidimensional scaling (NMDS) plots.** Samples were obtained from seawater and biofilms on glass and plastic (i.e. LLDPE, OXO, PA, PET and PLA) deployed for up to 12 months in the marine environment of the Lighter Basin Marina, Tāmaki Makaurau-Auckland Viaduct Harbour, Aotearoa-New Zealand. (A and B) Colour and asterisks represent significance: yellow – *P* < 0.05 (*), orange – *P* < 0.01 (**) and red – *P* < 0.001 (***). (B) Permuted betadisper *P*-values are shown above the diagonal, and observed betadisper values are below. (C and D) A multivariate t-distribution was assumed when drawing the ellipses with the “stat_ellipse” function of ggplot2, representing the variance observed among each (C) plastic condition and (D) sampling depth, with confidence intervals of 95%.

**Figure S9 | Relative abundance (%) of CSS-normalised unassembled metagenomic reads from microbial communities in seawater and on biofilms (i.e. on glass and five non-aged and aged plastic polymers) classified to level 1 (right axis) and level 2 (left axis) metabolic pathways using the Kyoto Encyclopaedia of Genes and Genomes (KEGG) database.** Substrates were deployed for up to 12 months in the marine environment of the Lighter Basin Marina, Tāmaki Makaurau-Auckland Viaduct Harbour, Aotearoa-New Zealand. KEGG annotations were aligned to predicted open reading frames via Diamond BlastP. The number next to the solid substrate corresponds to the paddle depth (i.e. 1 is the top paddle, 2 is the middle paddle, and 3 is the bottom paddle).

**Figure S10 | CSS-normalised relative abundance (%) of genes previously reported to confer plastic degradation, with associated genus and reported plastic degraded, based on 70% BlastP percentage identity.** Plastic-degrading enzymes were obtained from PlasticDB (Gambarini *et al.*, 2022). Glass and plastics (i.e. LLDPE, OXO, PA, PET and PLA) were deployed for up to 12 months in the marine environment of the Lighter Basin Marina, Tāmaki Makaurau-Auckland Viaduct Harbour, Aotearoa-New Zealand. Bubbles with a red outline represent genes where individual reads have been identified as significantly indicative of a specific substrate type and time point (P > 0.05, IndVal analyses; Dufrêne & Legendre, 1997).

**References**

Abarenkov K, Zirk A, Piirmann T, Pöhönen R, Ivanov F, Nilsson HR & Kõljalg U (2023) UNITE general FASTA release for Fungi. version:18.07.2023. doi:10.15156/BIO/2938067.

Andrews S (2010) FastQC: A quality control tool for high throughput sequence data. version:0.12.1. [Online]. Available online at: <http://www.bioinformatics.babraham.ac.uk/projects/fastqc>.

Apprill A, McNally S, Parsons R & Weber L (2015) Minor revision to V4 region SSU rRNA 806R gene primer greatly increases detection of SAR11 bacterioplankton. *Aquatic Microbial Ecology* **75**: 129-137, doi:10.3354/ame01753.

Bates D, Mächler M, Bolker B & Walker S (2015) Fitting linear mixed-effects models using lme4. *Journal of Statistical Software* **67**: 1 - 48, doi:10.18637/jss.v067.i01.

Bengtsson-Palme J, Hartmann M, Eriksson KM, Pal C, Thorell K, Larsson DGJ & Nilsson RH (2015) Metaxa2: Improved identification and taxonomic classification of small and large subunit rRNA in metagenomic data. *Molecular Ecology Resources* **15**: 1403-1414, doi:10.1111/1755-0998.12399.

Bolger AM, Lohse M & Usadel B (2014) Trimmomatic: A flexible trimmer for Illumina sequence data. *Bioinformatics* **30**: 2114-2120, doi:10.1093/bioinformatics/btu170.

Callahan BJ, McMurdie PJ, Rosen MJ, Han AW, Johnson AJ & Holmes SP (2016) DADA2: High-resolution sample inference from Illumina amplicon data. *Nature Methods* **13**: 581-583, doi:10.1038/nmeth.3869.

Dufrêne M & Legendre P (1997) Species assemblages and indicator species: The need for a flexible asymmetrical approach. *Ecological Monographs* **67**: 345-366, doi:10.1890/0012-9615(1997)067[0345:SAAIST]2.0.CO;2.

Gambarini V, Pantos O, Kingsbury JM, Weaver L, Handley KM & Lear G (2022) PlasticDB: A database of microorganisms and proteins linked to plastic biodegradation. *Database* **2022:** baac008, doi:10.1093/database/baac008.

Ihrmark K, Bodeker IT, Cruz-Martinez K, Friberg H, Kubartova A, Schenck J, Strid Y, Stenlid J, Brandstrom-Durling M, Clemmensen KE, Lindahl BD (2012) New primers to amplify the fungal ITS2 region--evaluation by 454-sequencing of artificial and natural communities. *FEMS Microbiology Ecology* **82**: 666-677, doi:10.1111/j.1574-6941.2012.01437.x.

Katoh K & Standley DM (2013) MAFFT multiple sequence alignment software version 7: Improvements in performance and usability. *Molecular Biology and Evolution* **30**: 772-780, doi:10.1093/molbev/mst010.

Kozich JJ, Westcott SL, Baxter NT, Highlander SK & Schloss PD (2013) Development of a dual-index sequencing strategy and curation pipeline for analyzing amplicon sequence data on the MiSeq Illumina sequencing platform. *Applied and Environmental Microbiology* **79**: 5112-5120, doi:10.1128/AEM.01043-13.

Laroche O, Pantos O, Kingsbury JM, Zaiko A, Wallbank JA, Lear G, Thompson-Laing J, Audrezet F, Maday S, Doake F, Abbel R, Barbier M, Masterton H, Risani R, Smith D, Theobald B, Weaver L & Pochon X (2023) A spatio-temporal analysis of marine diatom communities associated with pristine and aged plastics. *Biofouling* **39**: 427-443, doi:10.1080/08927014.2023.2226069.

Letunic I & Bork P (2024) Interactive Tree of Life (iTOL) v6: recent updates to the phylogenetic tree display and annotation tool. *Nucleic Acids Research* **52**: W78-W82, doi:10.1093/nar/gkae268.

Martin M (2011) Cutadapt removes adapter sequences from high-throughput sequencing reads. *EMBnet Journal* **17**: 3, doi:10.14806/ej.17.1.200.

Martinez Arbizu P (2020) pairwiseAdonis: Pairwise multilevel comparison using adonis. version:0.4.1. Github.

McLaren MR & Callahan BJ (2021) Silva 138.1 prokaryotic SSU taxonomic training data formatted for DADA2. Zenodo. doi:10.5281/zenodo.4587955.

McMurdie PJ & Holmes S (2013) phyloseq: An R package for reproducible interactive analysis and graphics of microbiome census data. *PLOS One* **8**: e61217, doi:10.1371/journal.pone.0061217.

Oksanen J, Blanchet FG, Friendly M, Kindt R, Legendre P, McGlinn D, Minchin P, O’Hara RB, Simpson G, Solymos P, Stevens MHH, Szöcs E & Wagner H (2020) Vegan: Community ecology package. version:2.5-7. Available online at: https://CRAN.R-project.org/package=vegan.

Parada AE, Needham DM & Fuhrman JA (2016) Every base matters: Assessing small subunit rRNA primers for marine microbiomes with mock communities, time series and global field samples. *Environmental Microbiology* **18**: 1403-1414, doi:10.1111/1462-2920.13023.

Paulson JN, Stine OC, Bravo HC & Pop M (2013) Differential abundance analysis for microbial marker-gene surveys. *Nature Methods* **10**: 1200-1202, doi:10.1038/nmeth.2658.

Price MN, Dehal PS & Arkin AP (2010) FastTree 2 - Approximately maximum-likelihood trees for large alignments. *PLOS One* **5**: e9490, doi:10.1371/journal.pone.0009490.

R Core Team (2023) R: A language and environment for statistical computing. R Foundation for Statistical Computing. Vienna, Austria.

Theobald B, Risani R, Donaldson L, Bridson JH, Kingsbury JM, Pantos O, Weaver L, Lear G, Pochon X, Zaiko A, Smith DA, Anderson R, Davy B, Davy S, Doake F, Masterton H, Audrezet F, Maday SDM, Wallbank JA, Barbier M, Greene AF, Parker K, Harris J, Northcott GL & Abbel R (2024) An investigation into the stability and degradation of plastics in aquatic environments using a large-scale field-deployment study. *Science of the Total Environment* 917: 170301, doi:10.1016/j.scitotenv.2024.170301.

Wallbank JA, Lear G, Kingsbury JM, Weaver L, Doake F, Smith DA, Audrézet F, Maday SDM, Gambarini V, Donaldson L, Theobald B, Barbier M & Pantos O (2022) Into the plastisphere, where only the generalists thrive: Early insights in plastisphere microbial community succession. *Frontiers in Marine Science* **9:** 841142, doi:10.3389/fmars.2022.841142.

Wallbank JA, Doake F, Donaldson L, Kingsbury JM, Masterton H, Pantos O, Smith DA, Theobald B, Weaver L & Lear G (2025) Microbes with plastic-degrading and pathogenic potentials are present on plastics in the final polishing pond of a wastewater treatment plant. *Environmental Microbiome* 20(1): 80.

Wickham H (2016) ggplot2: Elegant graphics for data analysis. Springer-Verlag New York. Available online at: https://ggplot2.tidyverse.org.
